# It Is High Time Physicians Thought of Natural Products for Alleviating NAFLD. Is There Sufficient Evidence to Use Them?

**DOI:** 10.3390/ijms222413424

**Published:** 2021-12-14

**Authors:** Giovanni Tarantino, Clara Balsano, Silvano Junior Santini, Giovanni Brienza, Irma Clemente, Benedetta Cosimini, Gaia Sinatti

**Affiliations:** 1 Department of Clinical Medicine and Surgery, Federico II University Medical School of Naples, 80100 Naples, Italy; tarantin@unina.it; 2 MESVA Department, University of L’Aquila, Piazzale Salvatore Tommasi 1, 67100 L’Aquila, Italy; silvanojunior.santini@univaq.it (S.J.S.); giovanni.brienza@graduate.univaq.it (G.B.); irma.clemente@graduate.univaq.it (I.C.); benedetta.cosimini@graduate.univaq.it (B.C.); gaia.sinatti@graduate.univaq.it (G.S.)

**Keywords:** natural products, nutraceutics, NAFLD, animal models, RCTs

## Abstract

Non-alcoholic fatty liver disease (NAFLD) is the most common form of liver disease all over the world due to the obesity pandemic; currently, therapeutic options for NAFLD are scarce, except for diet recommendations and physical activity. NAFLD is characterized by excessive accumulation of fat deposits (>5%) in the liver with subsequent inflammation and fibrosis. Studies in the literature show that insulin resistance (IR) may be considered as the key mechanism in the onset and progression of NAFLD. Recently, using natural products as an alternative approach in the treatment of NAFLD has drawn growing attention among physicians. In this review, the authors present the most recent randomized controlled trials (RCTs) and lines of evidence from animal models about the efficacy of nutraceutics in alleviating NAFLD. Among the most studied substances in the literature, the following molecules were chosen because of their presence in the literature of both clinical and preclinical studies: spirulina, oleuropein, garlic, berberine, resveratrol, curcumin, ginseng, glycyrrhizin, coffee, cocoa powder, epigallocatechin-3-gallate, and bromelain.

## 1. Introduction

Non-alcoholic fatty liver disease (NAFLD) is a complicated disease impacted by the complex interplay of genetic, epigenetic, and environmental factors [1,2]. In addition, several lifestyle factors, such as sedentary lifestyle, westernized diet, and smoking, enhance NAFLD risk [3]. 

Unfortunately, mechanisms inducing/worsening NAFLD/nonalcoholic steatohepatitis (NASH) are until now far from being completely clarified [4]. Nevertheless, there are many lines of research that should be reckoned as highly plausible.

**Figure 1:**
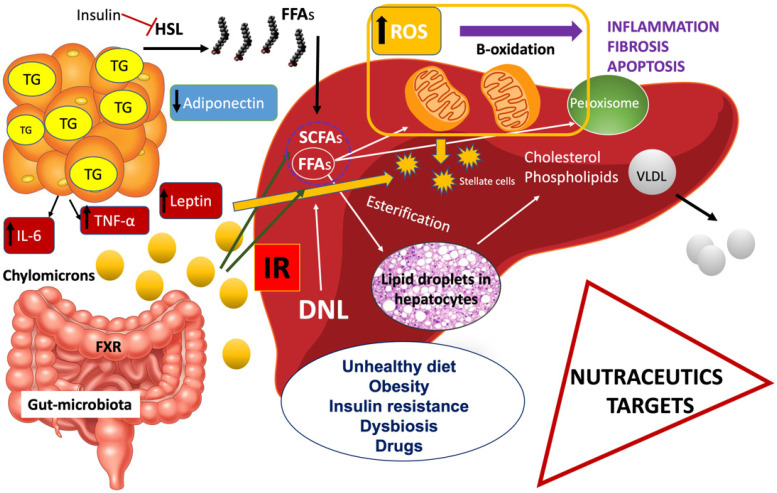
Principal pathophysiologic mechanisms in NAFLD. Insulin resistance (IR) is a multiorgan phenomenon. Additionally, adipose tissue and liver secrete proinflammatory cytokines. An unhealthy diet, obesity, insulin resistance, dysbiosis, and external factors such as drugs contribute to NAFLD progression.

The excessive lipid storage in the hepatocytes of NAFLD patients is represented by triglycerides (TG). The augmented influx of fatty acids (FFAs) derived from the diet, associated with de novo lipogenesis (DNL), and FFAs liberated from the adipose tissue contribute to accumulating TG in the liver, although not in a similar entity. FFAs stored in the liver and secreted via lipoproteins in NAFLD patients originate approximately 60% from adipose tissue, 25% from DNL, and 15% from the diet [5]. Accumulation of fat in the liver is associated with impaired insulin suppression of glucose production and serum FFAs [6]. FFAs are liberated by subcutaneous and visceral adipose tissue under the action of cytokines, such as tumor necrosis factor-alpha (TNF-a), interleukin-6 (IL-6), and interleukin-1b (IL-1b) [7], as well as leptin [8], while adiponectin (APN) plays a protective role in these molecular signals in the sense that it decreases elevated FFAs by oxidizing them in muscle [9,10] (Figure 1).

Elevated concentrations of FFAs cause peripheral and hepatic insulin resistance (IR) by inhibiting insulin-stimulated peripheral glucose uptake. Two mechanisms are responsible: (a) a fat-related inhibition of glucose transport or phosphorylation and (b) a decrease in muscle glycogen synthase activity. Interestingly, FFAs stimulate insulin secretion [11] (Figure 1).

Continuing to prove the theory that extrahepatic tissue contributes to liver disease; we should mention the key role of the small intestine. The gut microbiota as a mechanism inducing NAFLD has been receiving utmost interest from researchers. Increased intestinal permeability is related to obesity and NAFLD, and researchers are still debating whether this alteration represents an origin or an effect of disease [12]. Obesity and other metabolic dysfunctions associated with obesity are identified by peculiar transformations in the assembly/constitution and, consequently, the function of the human gut microbiota. These impairments are linked to decreased microbiome diversity (relative abundance of Firmicutes to the cost of Bacteroidetes) [13], which can be affected by various components of the diet. Specifically, the fasting-induced adipocyte factor is a serum lipoprotein lipase inhibitor. and its elimination is central to the deposition of TG in adipocytes, a process likely produced by microbiota [14]. It should be emphasized that other results contradict previous findings regarding the contribution of various bacterial groups to the progress of obesity, pointing to the production of short-chain fatty acids (SCFAs) [15].

It is noteworthy to stress that FFAs have a wide range of antibacterial activity comprehending lysis and solubilization of bacterial cell membranes as well as interference of adenosine triphosphate (ATP) production [16]. Furthermore, increased lipopolysaccharide production, also termed “metabolic endotoxemia”, may play an important role in obesity and related diseases such as NAFLD, due to being associated with an increased pro-inflammatory and oxidant environment, thus representing a key mediator of metabolic derangements observed in obesity [17]. Still, secondary bile acids, trimethylamine, and pro-inflammatory factors, i.e., the well-known lipopolysaccharide, may negatively impact hepatic lipid metabolism mediating the production of SCFAs [18].

Finally, the chemical modification of bile acids plays a further role in modifying lipid metabolism [19]. Bile acids activate the farnesoid X receptor (FXR) in the liver and through the enterohepatic circulation repress bile acid synthesis. Obesity and type 2 diabetes mellitus (T2DM) are both combined with decreased FXR activity and impaired metabolism of bile acids, with consequent alteration of hepatic lipid homeostasis and, what is more important, of insulin sensitivity [20].

## 2. Changes in Mitochondrial Function

Mitochondrial dysfunction is an important mechanism giving place to NAFLD and the more critical spectrum, i.e., NASH. Overload of FFAs or conditions inducing hyperglycemia produces increased reactive oxygen species (ROS) and reduces mitochondrial biogenesis, prompting mitochondrial dysfunction that, in turn, gives place to both decreased β-oxidation and ATP production, as well as further increased ROS production, in a vicious circle, eventually resulting in IR, central to NAFLD. Genetic factors related (mt-CYB, POLG, HSD17B13) or not (PNPLA3, GCKR, TM6SF2, MBOAT7) to mitochondria could impact this phenomenon [21,22,23,24].

Hepatic mitochondrial DNA (mtDNA) in NAFLD patients has been demonstrated to host complex genomes with a mutation rate and a heteroplasty grade higher (1.28 times) than normal ones [25]. The mitochondrial genome is particularly prone to various mutagenic stressors because mitochondrial genes are more adjacent to ROS provenance and are not preserved by histones. The mitochondrial respiratory chain is the main ROS subcellular source, which can damage mitochondrial proteins, lipids, and mtDNA [26].

Studies have shown that the intake of FFAs, which modifies the mitochondrial membranes and causes the production of ROS and damage to nearby structures, ultimately leading to inflammation, apoptosis, and progression of NAFLD [27].

Moreover, IR is intertwined with a decreased number of mitochondria, abnormal morphology, lower levels of mitochondrial oxidative enzymes, and lower ATP synthesis in human muscle biopsies [28]. These abnormalities comprehending depletion of mtDNA, reduced activity of respiratory chain complexes, and impaired mitochondrial β-oxidation are connected to the progression of NAFLD through NASH [29]. Mitochondrial biogenesis is propelled by peroxisome proliferator-activated receptor co-activator (PGC)-1, a transcriptional regulator of uncoupling protein (UCP) that is deeply involved in the insulin/gluconeogenesis signaling pathway and plays an important role in thermogenesis in adipose tissue [30].

A further key factor regulating mitochondrial biogenesis is adenosine monophosphate-activated protein kinase (AMPK) [31]. With aging fat mass, mainly visceral adiposity is disposed to steady augment, and both daily energy expenditure and physical activity are inclined to become lower since regulation of energy production is dependent on ATP needs. This process leads to decreased oxidative capacity in skeletal muscles [32].

As previously emphasized, due to the complexity of NAFLD pathogenesis, drug options for this very common disease are very poor. A different and more healthy diet combined with increased physical activity and supplemented by plant elements and extracts containing natural substances is considered useful and safe in order to reduce excess liver fat and decrease the risk of the progression to more severe liver disease.

## 3. Clinical Trials and Studies in Animal Models

Interestingly, there are several lines of research that have ascertained a likely therapeutic effect of natural products on NAFLD.

Many promising drug candidates are present in the current development pipeline that are of natural origin. We chose to select ongoing studies concerning natural products performed in both animal models of NAFLD and in patients suffering from NAFLD with the aim to show the utility of these compounds.

### 3.1. Alga Spirulina Maxima

Spirulina maxima is a cyanobacterium characterized by a gross content of proteins comprehending essential amino acids and by other factors, including the vitamin B complex associated with various minerals, as well as carotenoids, gamma-linolenic acid, and omega-3 and omega-6 fatty acids [33]. A pilot study, implanted to determine the effects of Spirulina on 55 Cretan patients with NAFLD, orally supplemented with 6 g of this dietary supplement per day, showed at the end of the 6-month intervention period that the mean levels of AST, ALT, gamma-glutamyl-transpeptidase (gamma-GT), triglycerides (TG), LDL-C, total cholesterol (TC), and the ratio of TC to HDL-C were significantly decreased. More interestingly, a significant reduction in weight and HOMA-IR index was found. Unfortunately, no modifications in sonographic features were observed [34]. Three Hispanic Mexican patients were treated with 4.5 g/day of spirulina maxima for 12 weeks; it is interesting that these patients showed a decrease in TG, TC, LDL-C, and TC/HDL ratio. Two of them showed a reduction in parenchyma heterogeneity when ultrasonography was performed, while the third patient showed a complete resolution of the “brilliant liver”, compared with before treatment with ultrasonography [35] (Table 1 and Table 2).

### 3.2. Olive Oil

Olive oil has been reckoned to have a protective effect on the cardiovascular (CV) system, impacting obesity, type 2 diabetes mellitus (T2DM), and related metabolic disorders [36].

A double-blinded RCT was conducted on 66 NAFLD patients, randomized into two groups, and 20 g/day of either olive oil or sunflower oil for 12 weeks was administered. A hypocaloric diet (nearly 500 kcal/d) was recommended to all participants. The following parameters were examined before and after intervention: fatty liver severity, liver enzymes, anthropometric parameters, blood pressure, serum lipid profile, glucose, insulin, malondialdehyde, total antioxidant capacity, and IL-6. Olive oil only decreased serum AST. Serum TG and fat mass significantly decreased after the ingestion of olive oil. Changes in fatty liver damage grade, as well as in skeletal muscle mass, were most important in subjects who were in the olive oil group, although the trials reported no modifications in body fat percentage [37]. Indeed, the beneficial effects of the Mediterranean diet on human health have been mainly attributed to its high content of extra virgin olive oil [38].

Santini et al. demonstrated that oleuropein (Ole) is able to improve the pro-inflammatory and antioxidant defense status in a murine model of NAFLD [39]. Moreover, oral administration of Ole in C57BL/6J mice, fed with an unhealthy diet, induced activation of autophagy characterized through AMPK-dependent phosphorylation of ULK1 at Ser555, regardless of the sex [40] (Table 1 and Table 2).

### 3.3. Garlic

In a recent RCT, 90 NAFLD patients were assigned to take either a garlic powder supplement (1600 mg) or a placebo for 12 weeks. At the end of the study, features of hepatic steatosis were significantly reduced in the treatment group, compared with the control group. Specifically, ALT, AST, and gamma-GT, but not ALP levels, significantly decreased, similar to TC, TG, and LDL-C, which also decreased in the treatment group, compared with the control group [41]. The same NAFLD population, in a parallel study, revealed a reduction in HOMA-IR, as well as a significant increase in skeletal muscle mass, serum concentration of superoxide dismutase, and total antioxidant capacity in the treatment group [42].

An insulin-resistant mouse (ddY-H), a mouse model of NAFLD, showed improved glucose intolerance and reduced hepatic TG accumulation when treated with garlic extract. Additionally, the intestinal microbiota pattern showed a better condition [43] (Table 1 and Table 2).

### 3.4. Berberine

Berberine (BBR) is reckoned as an alkaloid extracted from plants such as European barberry, goldenseal, and goldthread [44]. A parallel, open-labeled RCT was implanted enrolling patients from three investigation centers. In total, 184 patients suffering from NAFLD were studied and randomly received (1) lifestyle intervention (LSI), (2) LSI plus pioglitazone (PGZ) 15 mg qd, and (3) LSI plus BBR 0.5 g, respectively, for a period time of three months and a half. The authors, interestingly, offered evidence of hepatic BBR content and examined the expression of genes related to glucose and lipid metabolism in an animal model of NAFLD, to which BBR was successively administered. With respect to LSI, the combination of BBR plus LSI ended in a significant reduction in high-fat content (52.7% vs. 36.4%). To this effect followed a consistent recovery in body weight and an improvement in homeostasis model assessment of insulin resistance (HOMA-IR) and serum lipid profiles. BBR only was more functional than PGZ 15 mg qd in lessening body weight and ameliorating lipid profile. It is necessary to highlight the fact that adverse events, likely associated with BBR administration, were mild and affected mainly the digestive system [45].

Again, 35 Sprague Dawley rats were randomly split into the NAFLD group and the control group that was fed a normal diet for two months. The rats treated with BBR presented reduced liver wet weight, with ameliorated liver steatosis and a significant decrease in liver TG, ALT, AST, TC, TG. Notably, LDL levels significantly diminished. This effect was coupled with the significant upregulation of microsomal triglyceride transfer protein (MTTP), with increased levels of the same. All these findings were not present in the saline-treated NAFLD rats. Interestingly, BBR can cause adverse effects, including unexpected and not convenient interactions with prescription drugs, due to interference with the CYP2D6 and CYP3A4 enzymes, which are implicated in the biotransformation of endogenous compounds and xenobiotics [46,47] (Table 1 and Table 2).

### 3.5. Resveratrol

Resveratrol is a polyphenolic compound naturally found in peanuts, grapes, red wine, and some berries. In a double-blind, placebo-controlled RCT, 60 subjects with NAFLD were given two placebo capsules (placebo group) or 300 mg resveratrol capsules (resveratrol group) twice daily for three months. Compared with the placebo group, resveratrol significantly decreased GPT, glucose, and LDL-C, TC, and HOMA-IR. In the resveratrol group, significant reductions in the levels of TNF-a, cytokeratin 18 fragments, and FGF-21 and elevation of APN level were observed [48].

A crossover randomized double-blind study was led, including 44 youth adults, divided into a group intaking 250 mL of bayberry juice twice daily for 4 weeks, and a placebo control group. The first one showed decreased plasma levels of TNF-a and IL-8, proving inhibition in inflammatory and apoptotic response involved NAFLD. Additionally, an increased plasma antioxidant status and HDL-C level were detected [49] (Table 1 and Table 2).

### 3.6. Curcumin

Curcumin (Cur) belongs to the Curcuma longa species and it is highly present in Zingiberaceae, a member of the ginger family, as well as the turmeric.

NAFLD patients with different grades of disease were enrolled in an RCT, and 1 g/day of Cur was administered for 8 weeks. Supplementation with Cur was associated with a significant reduction in body max index (BMI) and waist circumference in the curcumin and placebo groups. Ultrasound analysis displayed a significant improvement in 75.0% of patients treated with Cur respect to the 4.7% of the control group. Serum levels of ALT and AST significantly slowed down only in the Cur group.

The authors found the Cur administration significantly reduced TG, LDL-C, fasting blood glucose (FBG), HOMA-IR, body weight, and AST levels. However, the observed decrease in TC, HbA1c, ALT, and insulin levels by Cur was not significant [50].

Authors of another study, very recently, performed a preclinical study on mice fed, for 10 weeks, a high-fat diet (HFD) or a normal diet supplemented or not with 0.2% Cur. The administration of Cur improved body fat, liver steatosis, insulin resistance and LPS serum levels. Interestingly enough, the related-Cur effects were appreciated also on the gut microbiota composition; in fact, the ratio of Firmicutes/Bacteroidetes and endotoxin-producing Desulfovibrio bacteria were decreased, whereas Akkermansia population and SCFA-producing bacteria were increased. These last bacterial genera altered by Cur were already reported to be correlated to the metabolic parameters in HFD-fed mice [51] (Table 1 and Table 2).

### 3.7. Ginseng

Many types of this herb are reckoned, but the most renowned ones are American ginseng (Panax quinquefolium) and Asian ginseng (Panax ginseng). In total, 80 patients with NAFLD were prospectively randomized to receive a three-week route of Korean red ginseng (KRG) or placebo. KRG was effective, in overweight patients with NAFLD, in restoring liver functional parameters, as well as in decreasing fat-related cytokines and molecules with antioxidant activity, whereas APN levels were increased [52] (Table 1 and Table 2).

### 3.8. Glycyrrhizin

Glycyrrhizin (GL) is the main bioactive element of licorice root. In a double-blind RCT, 66 NAFLD patients were enrolled and were separated into two groups: (i) treated group received 2 g aqueous licorice root extract per day for 2 months and (ii) placebo-control group. The authors found that GL administration significantly reduced ALT and AST serum levels, whereas the BMI did not significantly change in both groups [53]. The most important GL-related side effects were: hypertension and hypokalemic-induced secondary disorders [54].

Additionally, authors of another study conducted a preclinical study on 32 male Wistar rats randomly divided into (1) control group, fed a normal diet; (2) high-cholesterol diet (HCD) group; (3) normal diet plus GL 20 mg/kg; (4) normal diet plus GL 100 mg/kg, respectively, for 12 weeks. Interestingly, GL treatment at both doses, and especially at 100 mg/kg, significantly decreased levels of uncoupling protein 2 (UCP2) gene expression, which is involved in the decrease in ROS production by mitochondria [55] (Table 1 and Table 2).

### 3.9. Coffee

In a prospective, cross-sectional study, 1998 NAFLD patients were studied. Coffee drinking was categorized into no (0), moderate (1–2), and frequent (≥3) consumption (in cups/day). Most frequent coffee consumers (≥3 cups per day) had an inverse correlation with BMI, waist circumference, T2DM, liver enzymes, HOMA-IR, controlled attenuation parameter (CAP), and liver stiffness, in contrast with those who consumed 1–2 cups of coffee per day. In contrast, the female gender positively correlated with HDL-C [56].

Coffee intake reduced hepatic fibrosis in NASH patients. A validated questionnaire was used to assess for a relationship between caffeine and four groups: ultrasound negative (controls), light steatosis/not-NASH, NASH stage 0–1, and NASH stage 2–4 [57].

Furthermore, the authors studied the inverse correlation between coffee intake and the risk of NAFLD on C57BL/6 mice. Mice were treated, for 12 weeks, with a high-fat diet (HFD) or a normal diet supplemented or not with decaffeinated coffee. The coffee intake reduced liver steatosis beyond reducing transaminases and improved the oxidation of FFAs by the upregulation of acyl-CoA oxidase1 (ACOX1). Interestingly, the related coffee effects were also observed in its improvement of gut barrier function [58] (Table 1 and Table 2).

### 3.10. Cocoa Powder

Recent studies revealed that the consumption of cocoa powder, derived from Theobroma cacao, has a positive correlation with reduced risk of CV and metabolic diseases. However, the mechanisms of its hepatoprotective role on NAFLD were investigated only in limited studies.

Dark chocolate consumption is associated with a decrease in lipid peroxidation. A total of 100 subjects with T2DM were enrolled in an RCT and randomly assigned to the cocoa group (*n* = 50; received 10 g cocoa powder) or placebo group (*n* = 50), for 6 weeks. Cocoa consumption aimed to show probable interactions with prostaglandin synthase-2 (PTGS-2/COX-2), and it significantly decreased TG, LDL-C, HDL-C, TNF-α, and IL-6 [59].

The key mechanism at the basis of clinical benefits of dark chocolate is represented by its polyphenolic compounds, through the ability to inhibit the activity of nicotinamide adenine dinucleotide phosphate-oxidase (NADPH), which is the major source of oxidative stress [60,61].

Among possible side effects, chocolate has been implicated in conditions, such as acne and gastroesophageal reflux disease. Overall, the benefits of moderate cocoa consumption likely outweigh the risks [62].

In total, 19 NASH patients were enrolled in a cross-sectional study and separated into two groups of patients who took 40 g/day of dark chocolate (>85% cocoa) or 40 g/day of milk chocolate, for 2 weeks. The study demonstrated improvement of oxidative stress, which was evaluated by the activity of NOX2 and F2-isoprostanes, whereas hepatocyte apoptosis by cytokeratin-18 (CK-18) levels [63].

A study by Sun et al. examined the hepatoprotective effects of 80 mg/g cocoa powder supplementation for 10 weeks in HFD obese male mice. Cocoa induced an important antioxidant response and mitochondrial biogenesis, ameliorating hepatic oxidative stress and liver steatosis [64] (Table 1 and Table 2).

### 3.11. Green Tea

Epigallocatechin-3-gallate (EGCG), the most abundant catechin in green tea, has antioxidant, anti-carcinogenic, anti-hypertensive, and anti-fibrotic properties [65].

A double-blinded RCT has demonstrated that ingestion of a green tea beverage enriched with catechins with an EGCG -HFD reduced body weight (BW) in 126 obese adult patients. The patients were divided into the placebo, low-dose, and high-dose groups.

BW decreased significantly in the low-dose group and in the high-dose group [66].

The gut microbiota and their metabolites abnormalities are increasingly indicated to be at the bases of NAFLD. In fact, gut microbes produce SCFAs, hydrogen peroxides, trimethylamine, and ammonia. In recent years, several metabolites produced by microbiota have been shown to control lipid, carbohydrate homeostasis, and energy homeostasis in both extrahepatic and hepatic tissues. Oral administration of EGCG in mice fed HFD has effects on the gut microbiota, serum bile acid profile, and gene expression. EGCG significantly improved liver steatosis and intestinal dysbiosis [67] (Table 1 and Table 2).

### 3.12. Bromelain

Bromelain is extracted from stems of pineapples but is present in all parts of the fresh pineapple. As it is a concentrate of proteolytic enzymes, it may enhance anticoagulant activity (60). In an up-to-date study, HFD mice were treated or not with bromelain (20 mg/kg) for 12 weeks. Bromelain improved BW by ~30%, liver weight ~20%, and adipose tissue ~40%. The pathogenic mechanisms seem to be due to the reduced uptake of FFA by the intestinal wall and the better lipoprotein internalization. Moreover, the bromelain treatment increased bile acid metabolism, cholesterol clearance, the assembly and secretion of very-low-density lipoprotein (VLDL), and the β-oxidation of FFAs [68].

Bromelain treatment in 24 rats ameliorated the non-surgical treatment of periodontitis decreasing TNF-a. It was also able to reduce cholesterol, TG, ALT, and AST [69] (Table 1 and Table 2).

## 4. Criticism

Many studies presented in this review are consistent with the positive effects of natural products on histology features/laboratory data characteristic of NAFLD, but it should be highlighted that animal models of NAFLD do not completely mirror the human NAFLD.

Furthermore, no single animal model has encompassed the whole spectrum of human NAFLD, mainly when dealing with the more severe and progressive form, i.e., NASH, although very important in discovering some basic molecular processes [70].

Moreover, animal models dealing with natural products do not permit understanding the complex process of drug–drug interactions, very frequent in subjects on various drugs due to their co-morbidities such as T2DM, hypertension, or CV diseases, and the altered drug metabolism capacity in NAFLD patients [71].

Finally, adverse events (AEs) associated with the multiple uses of natural products should be identified [72]. AEs have different causes, such as impurities, batch-to-batch variability, misidentification and/or labeling, and different source of used production materials. Unfortunately, classic reporting systems do not always gather sufficient data on adverse events.

Further research is mandatory to build up models that more accurately mimic the disease spectrum to provide an increased understanding of the inner mechanisms and consequently identify future correct therapeutic approaches [73].

## 5. Conclusions

Diet and lifestyle modification are the cornerstones of the therapy of NAFLD, although many drugs are on the verge of being licensed.

In this review, the authors presented both RCTs and lines of research on animal models suggestive of a possible therapeutical effect by natural products, even though conclusive evidence will be reached with larger sample size studies in different populations, mainly evaluating the possible AEs.

## Figures and Tables

**Table 1:** Characteristics of clinical studies taken into account to prove the efficacy of natural products on NAFLD.

Authors	Year	Study	Compound	Duration	HOMA-IR	Liver Enzimes	Lipids Profile	Imaging/Biopsy	Mechanisms
Mazokopakis EE	2014	Pilot study	Spirulina	6 months	↑	↑	↑	No changes	↓ IL-6 and TNF-a ↑ APN level
Ferrera A	2010	Case series	Spirulina	3 months	↑	↑	↑	↓sonographic pattern of fat liver infiltration	↑ fat oxidation
Sangouni AA	2020	RCT	garlic	12 weeks	↑	↑	↑	↓ liver volume at ultrasound	↓ intestinal absorption of TGs ↑ APN level
Sangouni AA	2020	RCT	garlic	12 weeks	↑	↑	↑	Not detected	↓ mitochondrial dysfunction, kupffer cells activation ↓ gene expression of oxidative stress indices
Hajiaghamohammadi AA	2012	RCT	Licorice	8 weeks	↑	↑	↑	No changes at liver ultrasound	↓ oxidative stress
Guo H	2014	RCT	Berries	4 weeks	↑	↑	↑	Not detected	↓ oxidative stress ↓↓ TNFa and IL-8
Parsaeyan N	2014	RCT	Chocolate	6 weeks	↑	↑	↑	Not detected	prostaglandin synthase-2 (PTGS-2/COX-2)
Katz DL	2011	Review	Chocolate					Not detected	↑NF-jB ↓ xanthene oxidase, NADPH-oxidase, tyrosine kinases, and protein kinases
Loffredo L	2016	RCT	Chocolate	2 weeks	↑	↑	↑	Liver ultrasound and biopsy not detected	↓ xidative stress
Mikolasevic I	2020	Prospective, cross-sectional	Coffee	2013–2019	↑	↑	↑	↓ liver volume at liver ultrasound	↑ the blockade of transforming growth factor β expression ↓ connective tissue growth factor
Molloy JW	2012	Retrospective, cross-sectional	Coffee	2010–2011	↑	↑	↑	↓HFC at liver utrasound	↑ UDP glucuronosyltransferases
Yan HM	2015	RCT	Berberine	16 weeks	↑	↑	↑	↓ HFC at 1H MRS	↑ Expression MTTP, CPT-1a and GCK
Chen S	2015	RCT	Resveratrol	12 weeks	↑	↑	↑	No differences	↓ TNF-a, CK-18, FGF21 ↑ APN level
Panahi Y	2017	RCT	Curcumin	8 weeks	↑	↑	↑	↓ Portal vein diameter and liver volume at liver ultrasound	AMP-activated protein kinase ac- tivation
Hong M	2016	RCT	Ginseng	3 weeks	↑	↑	↑	↓ HFC at liver ultrasound	↓ TNF-a ↑ APN level
Rezaei S	2019	RCT	Olive oil	12 weeks	↑	↑	↑	↓ HFC and liver volume at liver ultrasound	↑ enhance fatty acid oxidation ↓ fat deposition
Peluso I	2016	Rewiew	Epigallocatechin-3-gallate		↑	↑	↑		↑ inhibitory effect on α-glucosidase, maltase, amylase, lipase, MDR1, OAT and PCFT
Kobayashi M	2016	RCT	Epigallocatechin-3-gallate	12 weeks	↑	↑	↑	↓ HFT	↑ fat oxidation ↓ fat absorption

Legend: ↑, increased; ↓, decreased; NA, not applicable; HFC, hepatic fat content; MTTP, microsomal triglyceride transfer protein; CPT-1a, carnitine palmitoyltransferase-1; GCK, glucokinase; TNF-a, tumor necrosis factor; CK 18, cytokeratin 18; FGF 21, fibroblast growth factor 21; APN, adiponectin; SCFA, short-chain fatty acids; AMP, adenosine monophosphate; HepG2, human hepatoma G2; IL-1, interleukin-1; G-CSF, granulocyte stimulating factor; SOD2, superoxide dismutase 2; Akt, protein kinase B; ULK1, unc51-like kinase 1; FFAR1, free fatty acid receptor 1; PPAR-a, peroxisome proliferator-activated receptor alpha; ACOX1, acyl-CoA Oxidase 1, ZO1, zonula occludens protein 1; UDP- glucuronosyltransferase, uridine5′-diphospho- glucuronosyltransferase; PTGS-2, prostaglandin-synthase-2; NFjB, nuclear factor kappa b; NADPH, nicotinamide adenine dinucleotide phosphate; AKT, v-akt murine thymoma viral oncogene homolog; SREBP-1, sterol regulatory element binding protein-1; FASN, fatty acid synthase; IL-6, interleukin-6; IL-10, interleukin-10; MDR1, multi drug reactivity 1 gene; OAT, organic anion transporter; PCFT, proton-coupled folate transporter; NK, natural killer cells; SREBP-2, sterol regulatory element binding protein-2; LXRa, liver x receptor a; ABCA1, ATP-binding cassette transporter1; ApoA1, apolipoprotein A1; CYP7A1, cholesterol 7 alpha-hydroxylase; ABCG5, ATP-binding cassette subfamily G member 5; ABCG8, ATP-binding cassette subfamily G member 8; TGs, triglycerides; PGC-1a, peroxisome proliferator-activated receptor-gamma coactivator; PEPCK, phosphoenolpyruvate carboxykinase. LDLR, apolipoprotein B and low density lipoprotein receptor.

**Table 2:** Characteristics of preclinical studies taken into account to prove the efficacy of natural products on NAFLD.

Authors	Year	Study	Compound	Duration	HOMA-IR	Liver Enzimes	Lipids Profile	Imaging/Biopsy	Mechanisms
Khan Z	2005	Review	Spirulina		↑	↑	↑	Not detected	↑ Phagocytic activity ↑ NK, T- and B- cells
Maeda T	2019	RCT	garlic	7 weeks	↑	↑	↑	↓ fat cells at biopsy	↓ Fat accumulation ↓ insulin resistence
Vitaglione P	2019	RCT	Coffee	12 weeks	↑	↑	↑	↓ HFC	↑ FFAR-1, PPAR-a, ACOX1 and ZO-1 expression
Sum M	2021	RCT	Chocolate	7 weeks	↑	↑	↑	↓ fat cells at biopsy	↓ hepatic oxidative stress
Chen P	2021	RCT	Berberine	8 weeks	↑	↑	↑	↓ fat cells at biopsy	↓ expression of MTTP and LDLR
Santini S J	2020	RCT	Olive oil	16 weeks	↑	↑	↑	↓ fat cells at biopsy	↓ fat in HepG2 cells ↓ IL1-α and G-CSF ↑ SOD2 cytosol expression
Porcu C	2018	RCT	Olive oil	16 weeks	↑	↑	↑	↓ HFC	↑ Activation of Akt/ULK1 pathway
Nayto Y	2020	RCT	Epigallocatechin-3-gallate	12 weeks	↑	↑	↑	↓ HFT	↑ intestinal dysbiosis
Hu PA	2020	RCT	Bromeline	12 weeks	↑	↑	↑	Not detected	↓ MCP-1, IL-6 and resistin ↑expression of SREBP-1, SREBP-2, LXRα, ABCA1, apoAI, CYP7A1, ABCG5, ABCG8
Alves EH	2020	RCT	Bromeline	20 days	↑	↑	↑	↓ HFT	↓ neutrophil migration to sites of inflammation ↓ expression of COX-2

## Data Availability

Not applicable.

## Abbreviations

NAFLD: nonalcoholic fatty liver disease; TG, triglycerides; DNL, de novo lipogenesis; FFAs, free fat acids; IR, insulin resistance; SCFAs, short-chain fatty acids; FXR, farnesoid X receptor; NASH, nonalcoholic steatohepatitis; T2DM, type 2 diabetes mellitus; ROS, reactive oxygen species; PGC, peroxisome proliferator-activated receptor co-activator; UCP, uncoupling protein; BBR, berberine; LSI, lifestyle intervention; PGZ, pioglitazone; HFC, hepatic fat content; HOMA-IR, homeostasis model assessment of insulin resistance; CUR, curcumin; HFD, high-fat diet; BMI, body max index; ALT, alanine aminotransferase; AST, aspartate aminotransferase; LDL-C, low-density lipoprotein-cholesterol; HDL-C, high-density lipoprotein-cholesterol; KRG, Korean red ginseng; GL, glycyrrhizin; HCD, high cholesterol diet; UCP2, uncoupling protein 2; Ole, oleuropein; ND, normal diet; CAP, controlled attenuation parameter; LSM, liver stiffness measurements; CC, coffee caffeine consumption; ROS, reactive oxygen species; EA, ellagic acid; IL, interleukin; BW, body weight; BR, brown rice; RA, retinoic acid; PNPLA3, patatin-like phospholipase domain-containing protein 3; GCKR, glucokinase regulator; TM6SF2, transmembrane 6 superfamily member 2; MBOAT7, membrane bound O-acyltransferase domain containing 7; MT-CYB, mitochondrially encoded cytochrome B; POLG, DNA polymerase gamma catalytic subunit; APN, adiponectin; RCT, randomized, controlled trial; ATP, adenosine triphosphate; TNF, tumor necrosis factor; CV, cardiovascular; AMPK, adenosine monophosphate-activated protein kinase; VLDL, very-low-density lipoprotein.
